# On the path to translation: Highlights from the 2010 Canadian Conference on Ovarian Cancer Research

**DOI:** 10.1186/1757-2215-4-10

**Published:** 2011-06-23

**Authors:** Brigitte L Thériault, Trevor G Shepherd

**Affiliations:** 1 Ontario Cancer Institute, Princess Margaret Hospital, 610, University Avenue, Toronto, ON, Canada; 2 London Regional Cancer Program, 790 Commissioners Road East, London, ON, Canada

## Abstract

Ovarian cancer continues to be the most lethal of the gynaecologic malignancies due to the lack of early detection, screening strategies and ineffective therapeutics for late-stage metastatic disease, particularly in the recurrent setting. The gathering of researchers investigating fundamental pathobiology of ovarian cancer and the clinicians who treat patients with this insidious disease is paramount to meeting the challenges we face. Since 2002, the Canadian Conference on Ovarian Cancer Research, held every two years, has served this essential purpose. The objectives of this conference have been to disseminate new information arising from the most recent ovarian cancer research and identify the most pressing challenges we still face as scientists and clinicians. This is best accomplished through direct encounters and exchanges of innovative ideas among colleagues and trainees from the realms of basic science and clinical disciplines. This meeting has and continues to successfully facilitate rapid networking and establish new collaborations from across Canada. This year, more guest speakers and participants from other countries have extended the breadth of the research on ovarian cancer that was discussed at the meeting. This report summarizes the key findings presented at the fifth biennial Canadian Conference on Ovarian Cancer Research held in Toronto, Ontario, and includes the important issues and challenges we still face in the years ahead to make a significant impact on this devastating disease.

## Introduction

Held in Toronto from May 15^th ^- 18^th^, 2010 in partnership with the Society of Gynecologic Oncology of Canada (GOC) and Ovarian Cancer Canada (OCC), the 5^th ^Canadian Conference on Ovarian Cancer Research was uniquely dedicated to presenting a multidisciplinary aspect to ovarian cancer research. From its inception in 2002, the Conference has evolved from a meeting of a few dozen clinicians and scientists to the participation of close to two hundred researchers and trainees involved in all aspects of ovarian cancer research. The conference featured renowned Canadian and International researchers presenting in symposia ranging from biomarker discovery and validation, cells of origin and models of ovarian cancer to susceptibility, risk factors, novel therapies, and current and emerging clinical trials for ovarian cancer. Two exciting workshops brought together expert physicians and scientists and provided a national forum in which to discuss the latest challenges facing banking of tissue, blood and fluid specimens for research purposes and in the successful management of clinical trials as we enter the era of molecular medicine and targeted therapies. For full details on meeting content, please visit the official website: http://www.ccocr.org/.

### Biomarkers and Early Detection

It is well-recognized that tumour heterogeneity complicates patient prognostics either in response to standard therapy or when directing patients to more targeted molecular approaches. Importantly, tumor heterogeneity also affects the accuracy of cancer diagnostics especially for early detection. Gene expression across patients reflects tumour heterogeneity and within these studies some known epithelial ovarian cancer (EOC) biomarkers have emerged including CA125, mesothelin and HE4. However, the applicability of these and other putative markers for early detection of EOC continues to fall short. Another important consideration is that the majority of EOC patients present with late-stage disease, making it difficult to identify true disease biomarkers that rise early during disease pathogenesis. Patrick Brown (Stanford University) presented mathematical modeling to define the "window of opportunity" for early detection of occult cancers within BRCA1 mutation carriers undergoing prophylactic oopherectomy [1]. Based on his results, the occult period for serous EOC within BRCA1 carriers is approximately 5 years, with the malignant cells spending the majority of this time between *in situ *lesions and stage 2 disease. Unfortunately, based on further calculations, in order to achieve a meagre 50% sensitivity in early detection of EOC using an annual test, the diagnostic test would need to detect a 1.3 cm diameter lesion. In contrast, detection would have to be at the 0.4 cm stage to achieve a meaningful 50% reduction in patient mortality. Brown pointed out that the inherent problems with this mathematical modeling are the underlying assumption that all early lesions will progress, despite the fact that some never do and even others will regress. With the strong push within the EOC research community for an early diagnostic serum biomarker test, Brown postulated that due to the signal-to-noise problems of the current technologies, the lesion would have to be 2.84 cm to detect above normal, falling short from making a true impact on mortality due to EOC. Nonetheless, Brown has aided in further defining the problem we need to solve. It is likely that given the size constraints imposed by the early-growing EOC tumours, early detection methods will require cheaper, faster, safer and higher-resolution imaging not available at the present time as well as different classes of tumour-specific biomarkers, for example novel fusion transcripts.

Brown's work raises an important question as to whether current molecular characterization techniques have the required resolution for early detection of EOC. David Huntsman (University of British Columbia) provided important insight into the latest technologies applicable to EOC. Via the use of next generation sequencing technologies, his group analyzes individual transcript sequences and refrains from pooling, directly addressing the impact of tumour heterogeneity. The advantage of transcriptome sequencing is that expressed genes represent a much smaller proportion than the genome but changes relevant to disease phenotype can still be observed.

Huntsman's group integrates the data from among the exomes of normal tissues and tumour specimens, as well as RNAseq data. Furthermore, the importance of studying each EOC subtype individually, then comparing each one against the other was stressed. The impact of this research strategy is evident in Huntsman's recent study of adult granulosa cell tumours, which identified a pathognomonic mutation in the *FOXL2 *gene yielding close to 100% disease penetrance [2]. This rare tumour type, however, is unique among ovarian tumours in the adult population. The most prevalent histotype of EOC is high-grade serous carcinoma and may represent an opposite example whereby multiple molecular routes can generate the same fairly homogenous tumour type. These high-grade tumours almost invariantly possess p53 loss of function and 50% lack functional BRCA1 [3]. Huntsman proposed that genomic chromosomal instability is the key underlying mechanism to development of high-grade serous tumours. This has obvious therapeutic implications with regards to the clinical use of small molecule inhibitors targeted to poly-ADP ribose polymerase (PARP).

The importance of histotype considerations in the development of novel biomarkers and therapeutic targets for EOC was echoed by Mark Carey (University of British Columbia), who demonstrated how one can exploit differential protein signalling pathways to achieve subtype-specific therapeutic benefit. Using a reverse phase protein array of 220 samples comprised of clear cell, high-grade (HG) serous and endometrioid EOCs, Carey's group was able to identify differential protein expression, where specific upregulation of AKT, Foxo3Ap, p70S6p, and p110α in HG serous, Src in clear cell, and Rab 25 in endometrioid were observed. Estrogen receptor was upregulated in HG serous and endometrioid tumors, and with further validation, could represent a good target in HG serous EOCs.

### Etiology and prevention - cells of origin and stem cells

An ever-present issue with EOC is the difficulty of achieving early-stage detection, partly because the natural history of each histotype is still largely unknown. Immense efforts are invested into identifying the cells or tissues that give rise to EOC, where development of targeted diagnostic tests and screening strategies are postulated to provide the basis for novel therapeutics to eradicate EOC growth and resistance.

John Dick (University Health Network) provided a historical perspective of the development of the cancer stem cell hypothesis. Many tumours show functional and morphologic heterogeneity, where only a small proportion of cells within the tumour have the capacity to initiate and sustain tumour growth. These cancer stem cells (CSCs) are defined by their capacity for self-renewal, their pluripotency, and their ability to drive continued expansion of the tumour population [4]. Clinical implications of this theory have fuelled great interest and excitement in the research community, as this hypothesis postulates that targeting CSCs could fully eradicate even chemoresistant tumours. If CSC properties govern tumour formation, they could also predict response and patient outcomes and thus be readily applied to clinical management of cancer patients. However, some researchers question the overall applicability of the CSC hypothesis. Dick emphasized that when applying the CSC hypothesis to solid tumours, more work is needed to better understand how each tumor cell type functions to initiate and sustain growth in mouse models.

Another fundamental question that remains is the identification of reliable markers that differentiate ovarian cancer initiating cells (CICs) from the rest of the tumour population. Jocelyn Stewart (University of Toronto) isolated primary serous EOCs directly from patients, sorted for the stem cell surface marker CD133^+^, and injected in limited dilutions into the mammary fat pad of NOD/SCID mice. Interestingly, tumours formed from both the CD133^+ ^and CD133^- ^populations, suggesting that CICs may be heterogeneous among patients or with disease progression. These data are consistent with a hierarchical model of serous ovarian cancer, and implicate CD133 as a potential, but not obligate, marker for ovarian CICs.

The long-held view of the OSE as a source of most EOCs has been challenged with reports identifying the distal fallopian tube epithelium (FTE) as a substantial source of high grade serous ovarian cancers [5]. Christopher Crum (Harvard University), a major contributor to the fallopian tube origin theory, has developed the SEE-FIM protocol (Sectioning and Extensively Examining the Fimbriated end) whereby the distal fallopian tubes of BRCA positive women who have undergone prophylactic salpingectomy are examined at the microscopic level for precursor lesions [6]. A high percentage (80%) of otherwise asymptomatic at-risk women present with serous tubal intraepithelial carcinomas (STICs), small proliferating neoplastic secretory cells with a diffuse positive p53 signal and a high proliferation index [7]. Fallopian tubes of women with confirmed high-grade serous ovarian cancer were also examined, and STICs were indeed found. Crum's findings demonstrate that the fallopian tube fimbria is a susceptible tissue for high-grade serous ovarian cancers. This research could lead to the development of new screening strategies to recognize small tumours in the fallopian tube, and knowledge of the pathways involved in tubal neoplasia could provide new treatments to intercept these lesions before spread to the ovary.

With the goal of identifying predisposing molecular alterations, Alicia Tone (University of Toronto) compared gene expression profiles of microdissected FTE from normal women, *BRCA *mutation carriers, and women with fallopian tube serous carcinoma and ovarian serous carcinoma. Tone provided evidence that the glucocorticoid receptor-mediated anti-inflammatory response post-ovulation is lost in FTE cells deficient in *BRCA1 *and *DAB2 *tumour suppressors. This sustained pro-inflammatory environment could contribute to an increased propensity for malignant transformation in the FTE.

### Models of Ovarian Cancer for mechanistic and therapeutic studies

Crucial to the better understanding of EOC etiology and progression, the development and study of *in vitro *and *in vivo *models of EOC provides invaluable tools to identify important disease modifies. These models also provide means to evaluate strategies for prevention, detection and treatment of disease that can facilitate the transition from bench to bedside. Denise Connolly (Fox Chase Cancer Center) presented her studies of reciprocal regulation of Src and STAT3 in murine models of EOC (MOVCAR cells and MISRII-TAg mice). Her group found that shRNA or small molecule-mediated inhibition of one molecule led to the activation of the other. While inhibition of either STAT3 or Src via shRNA reduced EOC cell migration and invasion, inhibition of both Src and STAT3 resulted in further impairment of migration than observed by inhibition of either target alone. These results indicate that STAT3 and Src are reciprocally regulated and suggest compensatory signaling pathways may be engaged to suppress or circumvent the effect of targeted inhibition. These findings have unexpected and important implications with regard to the use of molecular targeted therapeutic agents.

Rohann Correa (University of Western Ontario) presented his work on the characterization of a non-adherent, spheroid culture system of primary EOC ascites where he observed that spheroid formation represents a dormant state of EOC cells. This result points to a novel means of survival of EOC cells while in suspension, and could be an important mechanism contributing to the survival, chemoresistance and subsequent intraperitoneal dissemination of malignant EOC ascites.

Although animal models cannot completely replicate the human condition, researchers strive to develop animal models considering all currently known influences on tumorigenesis, including the microenvironment. With the goal of developing such an animal model for EOC, Jim Petrik (University of Guelph) is studying the importance of the ovarian stroma in the onset and progression of EOC. His group showed that EOC cells lines grown in the mouse ovarian bursa and subjected to ovarian stromal influcences undergo a "reprogramming" including behavioural changes such as increased growth and migration, and accelerated tumor growth when reintroduced into the mouse, but also exhibit genomic changes including increased genomic instability. Furthermore, gene expression profiling revealed altered expression of cell motility and metabolism genes. Uncovering ovarian stromal influences may point to early events responsible for the development of EOC, thus leading to novel therapies targeting these early events.

### Targeted Therapies in Current and Emerging Clinical Trials

The standard treatment for EOC is conventional cytotoxic chemotherapy of platinum and taxanes, which has remained essentially unchanged over the last two decades. Although initially effective, most patients will develop resistance, begging the need for new alternatives and ushering in a new era of "molecular medicine" specifically targeting molecular pathways responsible for disease.

Alan Ashworth (Institute of Cancer Research, London UK) discussed synthetic lethal approaches to cancer therapy, where tumors could not only be classified according to site or histological subytpe, but according to underlying genetic instability. This classification could optimize treatment and direct new therapeutic strategies towards synthetic lethality. For example, defects in DNA damage pathways in certain tumors could confer sensitivity to specific DNA damaging agents. Case in point, tumors carrying BRCA1 and 2 mutations have lost normal BRCA function, and developed extreme sensitivity to PARP inhibitors [8]. PARP inhibition in these cells therefore induces tumor-selective cell death, thus termed synthetic lethality [9]. This approach has been highly successful in Phase II clinical trials for the treatment of BRCA positive, advanced breast and ovarian cancers [10]. However in many cancers including EOC, BRCA function can be compromised through other mechanisms (epigenetic) apart from mutation, conferring BRCAness. Ashworth's team has uncovered PTEN as a mediator of PARP inhibitor sensitivity, and a potential biomarker for BRCAness [11] that could potentially identify a larger number of patients to benefit from PARP inhibition.

Lisa Kellenberger (University of Guelph) presented her data proposing that anti-hyperglycemic drugs may offer novel treatment opportunities for ovarian cancer. According to the Warburg effect, cancer cells have altered and inefficient glucose metabolism, increasing energy demand in comparison to normal cells [12]. In two independent diabetic mouse models (streptozotocin injection for type I disease and *Akt2*-deficient mice for type II disease), high levels of blood glucose generated larger tumour volumes presumably due to increased tumour demand. Treatment of ovarian cancer cell lines with the oral anti-hyperglycemic drugs rosiglitazone and metformin significantly decreased cell viability and VEGF expression, suggesting that targeting glucose metabolism in ovarian cancer may target tumour viability as well as the microvasculature.

Janet Dancey (NCIC Clinical Trials Group) offered an expert opinion on targeted therapies and clinical trials, their current status and future directions. Recently, striking activity was seen in early phase trials for PARP inhibitors in BRCA-deficient EOCs and triple negative breast cancers [13]. Swift drug development can occur when [1] a pharmacologically sound drug effectively interacts with its target, [2] target activation is a significant contributor to the specific malignancies of trial patients and [3] there is a means for accurately identifying such patients.

Unfortunately, simple linkage of good drug, good target and good test has remained elusive for many agents. The challenge is to narrow the gap between cancer biology, drug, and biomarker discovery and evaluation. A clinically useful biomarker yields a result that will assist treatment decisions, and although biomarker "discovery" is accelerating, few biomarkers have demonstrated such clinical utility.

The concept of personalized medicine, matching treatments to patients, has generated great enthusiasm. However, trials designed to evaluate treatments are not optimally designed to test hypothesis-driven biomarker questions. The realization of personalized medicine for EOC patients will require an integrated strategy of characterization and validation, biomarker and diagnostic test evaluation and testing of pharmacologically sound targeted agents and combinations.

In addition to novel and improved therapeutics, there is also a pressing need for development of screening strategies in order to detect ovarian cancer at earlier stages, thus improving prognosis. Jessica McAlpine (University of British Columbia) presented her work using optical technologies for screening of the fallopian tube to identify precursor lesions, or **T**ubal **I**ntraepithelial **C**arcinomas (TICs) in women at risk for hereditary ovarian cancer. Using direct fluorescence visualization *ex vivo*, TICs were identified via changes in reflectance and autofluorescence signals at the lesion site which were confirmed by subsequent histopathology. This technique may provide a novel, easy-to-use and adaptable *in vivo *screening device for the future goal of discerning malignant from benign tumours, and early cancers in high-risk patients.

### Workshops

#### Epithelial Ovarian Cancer Biobanking: It's all about the deposits and withdrawals

Multiple biobanking trials and initiatives are currently underway or are in development within Canada, and a number of important ethical, regulatory and technical issues have been raised. The goal of this workshop was to reach a survey of the critical issues from a multidisciplinary audience, in the hopes of uncovering innovative solutions. Helen Mackay (University Health Network) and David Huntsman discussed the companion study to the OV21 clinical trial investigating the potential benefit of intraperitoneal chemotherapy. Study design involves the prospective collection of tumor tissue and peripheral blood samples at diagnosis, cytoreductive surgery (or biopsy following neoadjuvant IV chemotherapy) and at progression. Tissue collection would be linked to a prospectively acquired clinical database, providing a platform for high quality translational research.

An identified challenge is the acquisition of adequate tissue amount without compromising patient care. Minimally invasive procedures such as laparoscopic biopsies and microfluidic technologies were proposed, however the amount obtained from these biopsies may be insufficient for future analyses. It was agreed that the tissue collection team should be expanded to include radiologists, who would assist with imaging technologies. Another discussion point reflected on the joint efforts of the Terry Fox Research Initiative (TFRI) and the Canadian Partnership against Cancer (CPAC) to develop a National Biomarker Program in Ovarian Cancer. Led by Janet Dancey and Anne-Marie Mes-Masson (Institut du Cancer de Montréal), the project deliverables are to build a national biobank consisting of a retrospective well-annotated classification system of tissues, and to assemble a pan-Canadian team of researchers to collaboratively advance the discovery and validation of novel biomarkers for ovarian cancer. Phase I has been completed, with 9 hospital centers throughout the country collectively providing over 2000 retrospective tissues for initial biobank quality control analyses. Phase II of the project is in its infancy, and the discussion focused on criteria for prospective collection of samples and clinical data, the application process and eligibility criteria for researchers, the prioritization of diagnostic markers to test, the technology of the assay(s) for biomarker validation, and cost analysis to implement centers that do not normally perform biobanking.

#### Translating molecular targets to clinical trials

The discovery of molecular targets in epithelial cancers has fuelled the development of many targeted anticancer drugs; however, many such therapies have failed to achieve the anticipated clinical outcomes. Jeremy Squire (Queen's University) and Amit Oza (University Health Network) presented lessons learned from failed trials, and described challenges associated with targeted therapy clinical trials. Rudimentary biomarker analysis may lead to unreliable selection of a patient population, where patients unnecessarily treated. Selection criteria should include the biologic rationale for targeting the pathway of interest, the genomic/proteomic profile of the tumour, the histological subtype, the molecular activation of the target pathway, the type of tissue used for screening and imaging modalities.

A major challenge, however, is the availability of assays that are analytically and clinically valid and useful. Other considerations include the availability of reliable testing platforms country-wide, and the turnaround time for validated assays. Most likely larger hospital centers with more staff and resources may become "hubs" of testing for biomarker trials. The rich clinical data collected will need to be assimilated via a multidisciplinary approach involving information technology, systems biology, and statistics. Ethical issues were raised regarding the responsibility of researchers and clinician scientists towards access to potentially impactful patient data. A clear plan of action needs to be developed to appropriately address this eventuality before it becomes a reality. Thus to go forward successfully, a standardized, rigorous sample collection protocol must be implemented in all participating centres to fulfill the assay requirements.

## Concluding Remarks

The 5^th ^CCOCR Meeting accomplished far more than its stated objectives of providing a forum for the sharing and dissemination of the latest advances in ovarian cancer research and treatment. Building upon the momentum generated in the previous meetings, this conference has for the first time attracted international interest. The findings presented at this meeting have greatly added to the existing knowledge of ovarian cancer biology and potential therapeutic targets. However how this knowledge is translated into clinically effective therapies remains an immediate challenge. This meeting has renewed and strengthened the resolve of the Canadian ovarian cancer scientific and clinical research communities to continue a forum of open discussion between disciplines in order to face and effectively overcome the roadblocks on the path to translation.

## Competing interests

The authors declare that they have no competing interests.

## Authors' contributions

BT synthesized the information from the meeting, and designed and wrote the manuscript. TS synthesized information from the meeting, and facilitated in the design and writing of the manuscript. All authors read and approved the final manuscript.
